# Magneto-mechanically actuated microstructures to efficiently prevent bacterial biofilm formation

**DOI:** 10.1038/s41598-020-72406-8

**Published:** 2020-09-22

**Authors:** S. Leulmi Pichot, H. Joisten, A. J. Grant, B. Dieny, R. P. Cowburn

**Affiliations:** 1 Department of Physics, University of Cambridge, Cambridge, UK; 2 CEA, CNRS, Spintec, Univ. Grenoble Alpes, 38000 Grenoble, France; 3 Department of Veterinary Medicine, University of Cambridge, Cambridge, UK; 4 CEA, LETI, Univ. Grenoble Alpes, 38000 Grenoble, France

**Keywords:** Biofilms, Magnetic properties and materials, Actuators

## Abstract

Biofilm colonisation of surfaces is of critical importance in various areas ranging from indwelling medical devices to industrial setups. Of particular importance is the reduced susceptibility of bacteria embedded in a biofilm to existing antimicrobial agents. In this paper, we demonstrate that remotely actuated magnetic cantilevers grafted on a substrate act efficiently in preventing bacterial biofilm formation. When exposed to an alternating magnetic field, the flexible magnetic cantilevers vertically deflect from their initial position periodically, with an extremely low frequency (0.16 Hz). The cantilevers’ beating prevents the initial stage of bacterial adhesion to the substrate surface and the subsequent biofilm growth. Our experimental data on *E. coli* liquid cultures demonstrate up to a 70% reduction in biofilm formation. A theoretical model has been developed to predict the amplitude of the cantilevers vertical deflection. Our results demonstrate proof-of-concept for a device that can magneto-mechanically prevent the first stage in bacterial biofilm formation, acting as on-demand fouling release active surfaces.

## Introduction

Bacterial biofilms are commonly defined as complex heterogeneous systems, comprising an aggregate of bacteria lodged in a three-dimensional extracellular matrix. Unlike in the planktonic form, bacteria embedded in biofilms benefit from a greater defence against environmental and chemical stresses^1^. Once adherent to a surface, bacteria replicate and produce extracellular polymeric substances, forming microcolonies. The microcolonies grow to form biofilms that can cover the entire surface of a structure, causing for example, fouling of products^2^ and infections^3^.

The tendency of bacteria to form biofilms during an infection seriously complicates their eradication as most of the antibiotics dedicated to planktonic bacteria often fail to eradicate infections caused by bacteria growing in biofilms^4^. One reason being the difficulty of antimicrobial agents to diffuse inside the biofilm matrix. A second mechanism that explains reduced biofilm susceptibility to antimicrobial agents is the presence of slow-growing or non-growing cells due to the nutrient limitation in a biofilm environment, that transforms bacterial cells into persisters^5^. These bacteria have a limited susceptibility to many antimicrobial agents and can survive metabolically-directed challenge.

Bacterial biofilms mediate frequent infections on indwelling medical devices. As an example, biofilm-related infections have been reported for central venous catheters, mechanical heart valves and urinary catheters^6^. An important issue in the health sector is the undesirable formation of flexible three-dimensional filaments called streamers^7,8^, which can lead to the clogging of pipes in medical devices. Furthermore, repeated applications of cleaning cycles on medical devices and in industrial settings are not viable, as they have been shown to fail or to enhance the undesirable effect of promoting resistance of the bacteria. A previous study reported that a repeated cleaning of paediatric tracheostomy tubes with sodium hypochlorite and household detergent promotes *Staphylococcus aureus* biofilm development^9^. Another study^10^ reported that biofilms grown in a simulated pork processing environment showed persistence when challenged with both quaternary ammonium compound sanitizers and sodium hypochlorite.

In order to be efficient, antibiofilm innovations need to tackle multiple mechanisms simultaneously as biofilms exhibit multiple resistance mechanisms toward antibiotics^1^. Potential solutions have explored the use of biofilm matrix-degrading enzymes and a wide range of chemical reactions that interfere with biofilm matrix synthesis^11^.

The current literature supports the notion that it is the aggregation of bacteria into multicellular communities that confers antibiotic and/or antimicrobial resistance to bacterial communities constituting biofilms, as bacteria that are dispersed from biofilms rapidly revert to a susceptible planktonic phenotype^6^. Therefore, a rational strategy would be the development of a technology that disrupts the multicellular biofilm structure to restore the efficiency of antibiotics and/or antimicrobials towards the non-associated bacteria.

Engineered surfaces that have the ability to apply mechanical forces are another promising, yet poorly explored solution, whereby a device could be used to weaken existing biofilms or prevent the build-up of new ones. The mechanical removal aims to promote the release of either loosely attached cells, or disassemble the multicellular structure by overcoming the biofilm cohesive forces. A previous study reported the use of inflation-generated strain to remove biofilms^12^. Recent studies exploited the use of iron oxides nanoparticles to tackle biofilm formation^13–15^.

This paper describes the development and use of active surfaces which prevent bacterial growth and promote the detachment of newly formed bacterial biofilms. Our approach exploits the magnetic and mechanical properties of surface-grafted microstructures. Each microstructure has a free-standing magnetic component designed specifically for biofilm removal. When exposed to a variable magnetic field, the free-standing parts of the microstructure vertically deflect from their initial positions, which instantaneously applies a mechanical force to any attached bacteria, both repelling bacteria from the surface and locally dispersing any biofilm that has colonised the chip surface. In this paper, we hereafter refer to the free-standing magnetic part as a “cantilever”.

In our experiments, each cantilever has a thickness of tens of nanometres and has a micrometric length. As a proof-of-concept, the magneto-mechanical surfaces were evaluated for biofilm formation by the common laboratory bacterial species, *Escherichia coli* K-12 strain MG1655. The magneto-mechanical response of the magnetic cantilevers actuated by a magnetic field was investigated theoretically by a magneto-mechanical model.

The results presented in this paper demonstrate efficient bacteria-repelling surfaces using the remotely actuated magnetic cantilevers. We hypothesize that the observed effects is caused by mechanical repulsive forces, mediated through fluid-flow created by the oscillating magnetic cantilevers. Our data shows that remotely actuated devices could outperform antibiotics and antimicrobial drugs on devices colonised with bacterial biofilms. Importantly, this can be achieved without the prior need to know the bacterial species or strains forming the biofilm.

## Results

### Magnetic cantilevers actuation with an external magnetic field source

**Figure 1:**
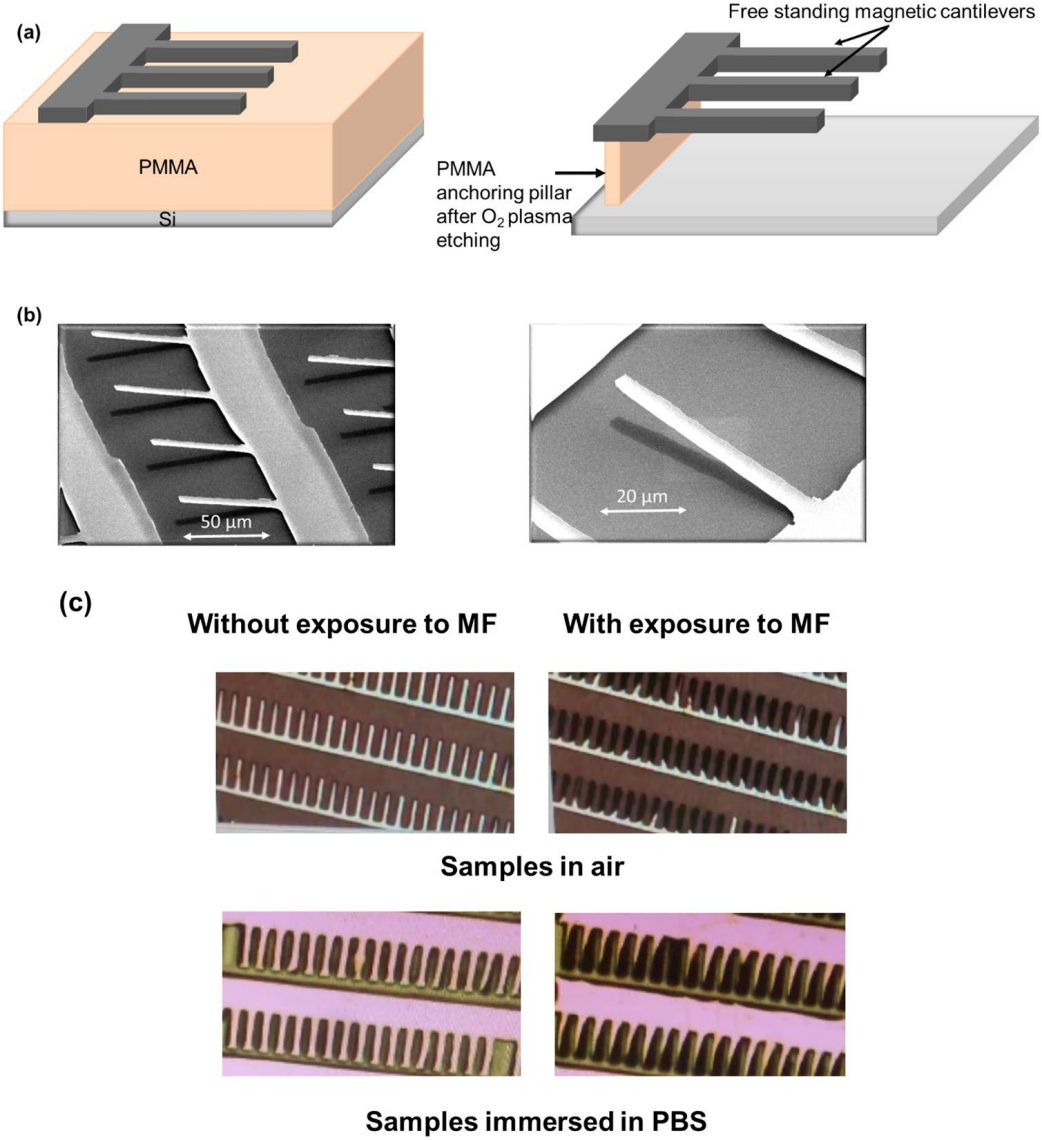
(**a**) Schematic of the magnetic cantilevers fabrication process. (**b**) SEM images of cantilevers at two magnifications. (**c**) Images taken during optical microscopy imaging of samples either in air (top) or immersed in PBS (bottom), in the presence or absence of a magnetic field source (neodymium iron boron (NdFeB) magnet). Both samples show a reflectivity change when exposed to the magnetic field (MF) as a result of their vertical deflection.

The magnetic cantilevers were fabricated by a top-down technique on silicon (Si) substrates. The substrate was first coated with a sacrificial layer of photoresist. The free-standing parts of the micrometric magnetic cantilevers were released from the substrate by an oxygen plasma etch that removes the underneath sacrificial photoresist. The cantilevers are linked to the substrate through a feature with wider dimensions to ensure that the oxygen plasma etches only the resist underneath the cantilever, allowing the attachment contact to the Si through the sacrificial photoresist pillar (Fig. 1). Bending can be observed on the free-standing part of the cantilevers after their release, which is due to the residual stress that builds-up during the multilayer deposition process^16^. Scanning electron microscope (SEM) images of arrays of magnetic cantilevers are shown in Fig. 1.

When exposed to a magnetic field (NdFeB permanent magnet), the cantilevers are deflected about a hundred of nanometers away from their initial position, generating a reflectivity change on the chip surface that is visible at a macroscopic scale to the naked eye. The magnetic cantilevers return to their initial position when the magnetic field is removed (Fig. 1c). Videos of the real-time deflection of the free-standing part of the cantilevers both in air (movie 1), and submerged in a phosphate-buffered saline (PBS) solution (movie 2), when exposed to a magnetic field are available in the supplementary information accompanying this paper. Similar observations have been previously reported by Truong et al.^17^.

We assessed various spacings between the cantilevers and evaluated the effect of these spacings on bacterial biofilm formation. Changing the spacing between each free-standing microstructure enabled us to investigate the influence that cantilevers oscillations have on the biofilm formation.

Cantilevers deflection measurements in response to the uniformly applied magnetic force.

Cantilevers actuation by the used magnet was modelled and the resulting vertical deformation was calculated using the Euler–Bernoulli beam theory^18^. The deflection calculation required successively the determination of the magnetic field amplitude and gradient applied by the magnetic source first, and then resulting magnetic forces exerted on the cantilevers.

**Figure 2:**
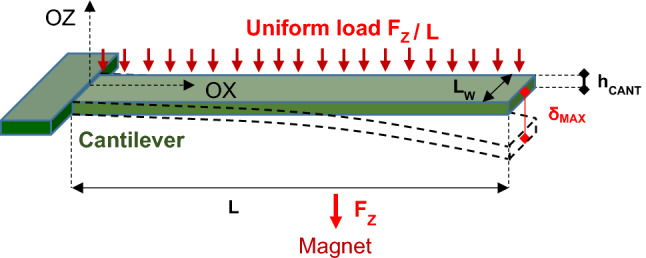
Sketch of the cantilever bending, submitted to the uniformly distributed magnetic force. Resulting δ_MAX_ = maximal deflection of the cantilever at its free extremity. Cantilever dimensions: Length L = 70 µm, width LW = 10 µm. Total thickness h_CANT_ = 144 nm. E is the approximate Young modulus of the metallic cantilever of composition Ta 2 nm, Pt 10 nm, CoFeB 50 nm, Ta 2 nm, CoFeB 50 nm, Au 30 nm, E ≈ 200 GPa.

The cantilevers are considered to be long, thin beams, made of homogeneous materials, with the deformation occurring in the (*zOx*) plane. The magnetic force exerted on the cantilevers is mostly uni-directional along the OZ axis. In the experimental configuration described in Fig. 2, the substrate is brought close to the magnet surface (2.5 mm away from the magnet surface), thus we consider BZ as the main active component of the field acting on the magnetic cantilevers. Any magnetic force parallel to the XY plane would not result in a substantial deformation as the cantilevers degree of freedom is essentially perpendicular to the plane and is considered as the most effective for the biofilm removal.

The cantilever is modelled as a clamped beam of length L, and of thickness h_CANT_ in the plane of bending^18^. Here, we assume, that this metallic multilayer cantilever is equivalent to a beam made of a homogeneous material. Its Young’s modulus is thus chosen with an average value E = 200 GPa, close to the Fe’s one.

In order to determine the cantilever deflection δ_max_ at the cantilever free extremity, we consider a unique cantilever composed of Ta 2 nm, Pt 10 nm, CoFeB 50 nm, Ta 2 nm, CoFeB 50 nm, Au 30 nm. The cantilever is placed above a stirrer-magnet at the distance Z, on OZ-axis of the cylindrical magnet surface.

The maximum deflection at the free end of the cantilever is expressed as a function of the magnetic force per unit length Fz (see methods for Fz calculation), and $E\cdot I$ characterizing the bending stiffness, as follows^19,20^:

1 $${\delta }_{MAX}=\frac{\left({F}_{Z}/L\right)\times {L}^{4}}{8\cdot E\cdot I}$$

where, for this cantilever of rectangular cross section, the moment of inertia $I$, determined by the cantilever width L_W_, and the thickness h_CANT_ only^21^, is expressed as:

2 $$I=\frac{{L}_{W}\times {\mathbf{h}}_{\mathbf{C}\mathbf{A}\mathbf{N}\mathbf{T}}^{3}}{12}$$

According to expression (1), the deflection scales as the fourth power of the cantilever length. Since the magnet size is much larger than the cantilever dimensions, the magnetic force per unit length (F_z_/L) is independent of the cantilever length L.

**Figure 3:**
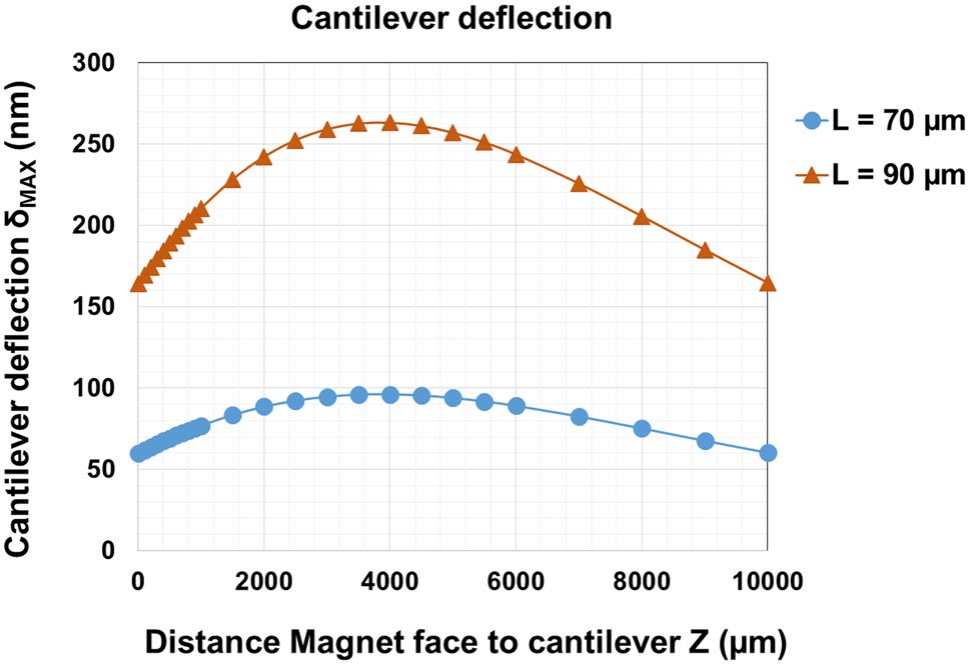
Maximal deflection δ_MAX_ at the free end of the cantilever, submitted to the magnetic force F_Z_ vs Z. The force F_Z_ = V. M(B))dB_Z_/dz is applied by the magnet (magnet thickness h = 5 mm, diameter DØ = 25 mm, of magnetization 1,18 T) on cantilevers of magnetization µ_0_M = 1.25 T, dimensions L (µm) × 10 µm × 100(144) nm, with L = 70 µm ; 90 µm. In particular at Z = 2,500 µm, δ_MAX_ = 92 nm and 252 nm respectively for L = 70 µm and L = 90 µm*.*

Based on the above expressions, Fig. 3 shows the cantilever deflection at its end as a function of the magnet-to-cantilever distance, having considered the magnetic state of the cantilever as saturated. The theoretical model developed above was also used to predict the amplitude of the vertical deflection reached by longer cantilevers, using the same experimental magnetic field source. A quantitative description of the relationship between the cantilever length (70 µm and 90 µm) and the corresponding deflection is given in Fig. 3.

Based on the magnetic properties of the cantilevers and the magnetic field source, the deflection of the free-standing part of each cantilever presents an optimum at about 3 mm above the magnet. This order of magnitude is calculated for a cantilever which is magnetically saturated. The calculations demonstrate that the setup used for this experiment triggers cantilever deflections of about 90 nm, for cantilevers having a length of 70 μm. The amplitude of this deflection is about one tenth of an individual *E. coli* bacterium diameter.

As shown by Eqs. (1) and (2), the longer the beam, the larger the vertical deflection. The deflection reaches values of δMAX = 92 nm and 252 nm respectively for L = 70 μm and L = 90 µm.

Our experimental data on *E. coli* liquid cultures (detailed in the section below) demonstrate a reduction in biofilm formation using actuated 70 µm long cantilevers. Changing the cantilevers length to 90 µm, thus increasing their vertical deflection, might be more efficient in repelling planktonic bacteria from the surface, hence inhibiting the biofilm growth. This hypothesis is yet to be further investigated experimentally.

### Magnetically driven bacteria-fouling surface

In a first attempt, we designed an experiment in which *E. coli* bacteria in a liquid culture are allowed to form an early stage biofilm on the substrate surface grafted with magnetic cantilevers. With the foreseen aim of breaking down the biofilm structure by applying a punctual magnetic actuation, that induces the cantilevers vertical deflection.

**Figure 4:**
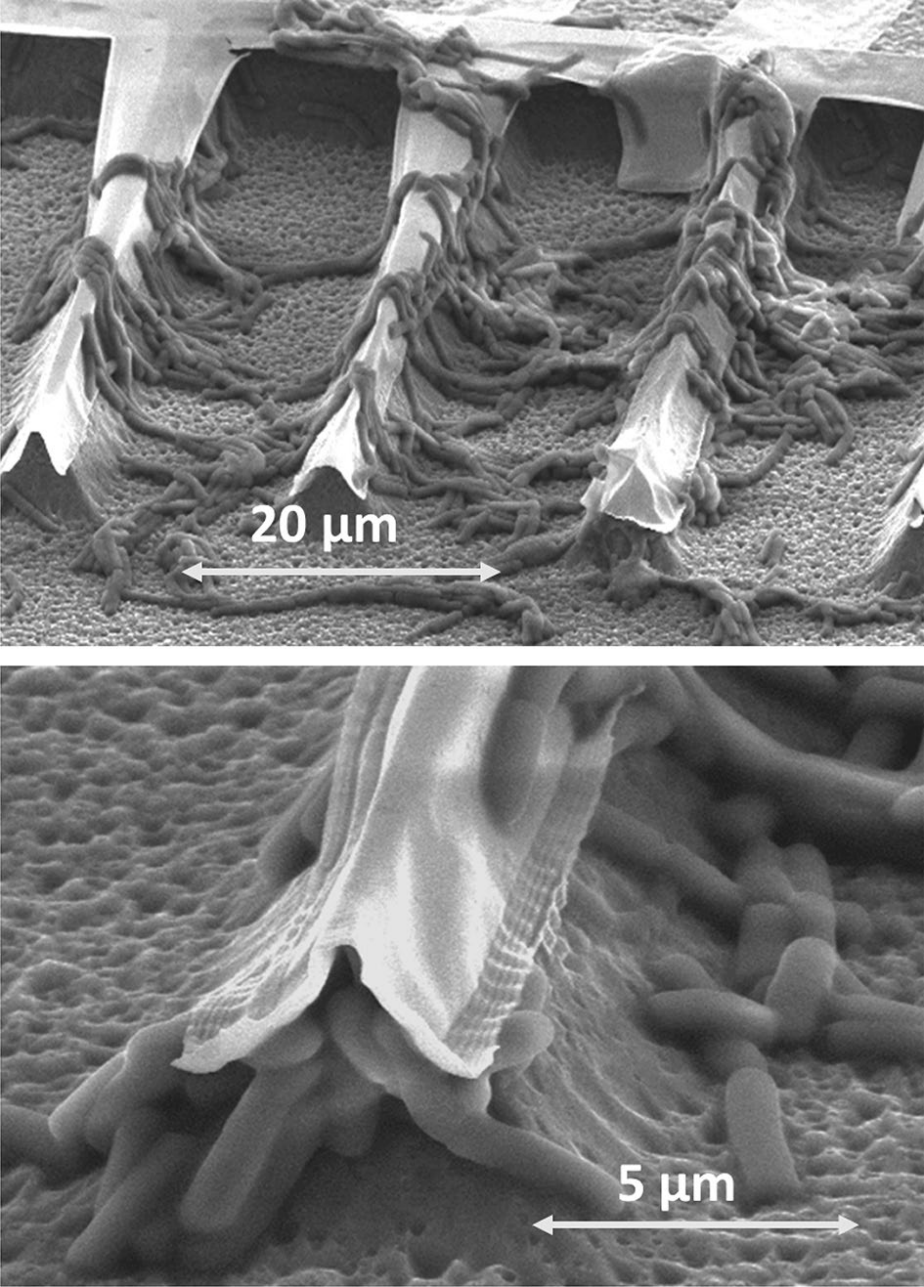
SEM pictures of magnetic cantilevers 24 h after inoculation with *E.coli* liquid culture showing the cantilevers collapse on the surface. The underneath pyramidal profiles are a result of the isotropic silicon etching. The isotropic etching was used in the first trials to release the free ends of the cantilevers from the substrate, using SF_6_ plasma etch.

Results of this initial set of experiments reveal a striking phenomenon. The adhesive forces developed by *E.coli* bacteria to anchor themselves to the substrate overcome the equilibrium forces maintaining the free ends of the cantilevers suspended. Which resulted in the cantilevers collapse on the substrate surface as shown in Fig. 4. Furthermore, the adhesive forces developed during the buildup of the bacterial biofilm overcome the magnetic forces acting on the cantilevers when applying a magnetic field. Indeed, 24 h after the inoculation with *E.coli* liquid culture, the cantilevers do not deflect from their initial position as expected when applying a magnetic field and seem firmly stuck to the substrate through the bacterial biofilm. It then appeared necessary to adjust the composition and shape of the cantilevers to find the best tradeoff between a sufficient mechanical stiffness to withstand the bacterial biofilm adhesion force without collapsing on the substrate while maintaining a sufficient flexibility to enable vertical deflection under the applied magnetic field. We emphasize that such optimizations require a dedicated investigation in a follow-on study, which would determine the materials and geometries to be adopted according to the biofilms characteristics (i.e. bacterial strain, biofilm maturity, growth conditions).

When used as a static surface, the substrate became non-actuable after the bacterial biofilm formation as shown above. Therefore, we used our substrate as a dynamic surface to assess the biofilm formation, by applying the magnetic field that induces the cantilevers oscillations as soon as the substrate was inoculated with the bacterial suspension. The cantilevers beat with a permanent periodic oscillation at a very low frequency (0.16 Hz).

Following 48 h of *E. coli* MG1655 biofilm growth, bacterial microcolonies evolutions were observed by optical microscopy at various locations on the substrates. Each substrate included a patterned area that was depleted from cantilevers that served as an internal magnetic field control for each chip—*i.e.* the bacterial suspension experiences no fluid stirring due to oscillating cantilevers, but the area remains exposed to the same magnetic field and the substrate went through the same chemical processes during fabrication as the area of interest; including solvent lift-off and oxygen plasma etch.

**Figure 5:**
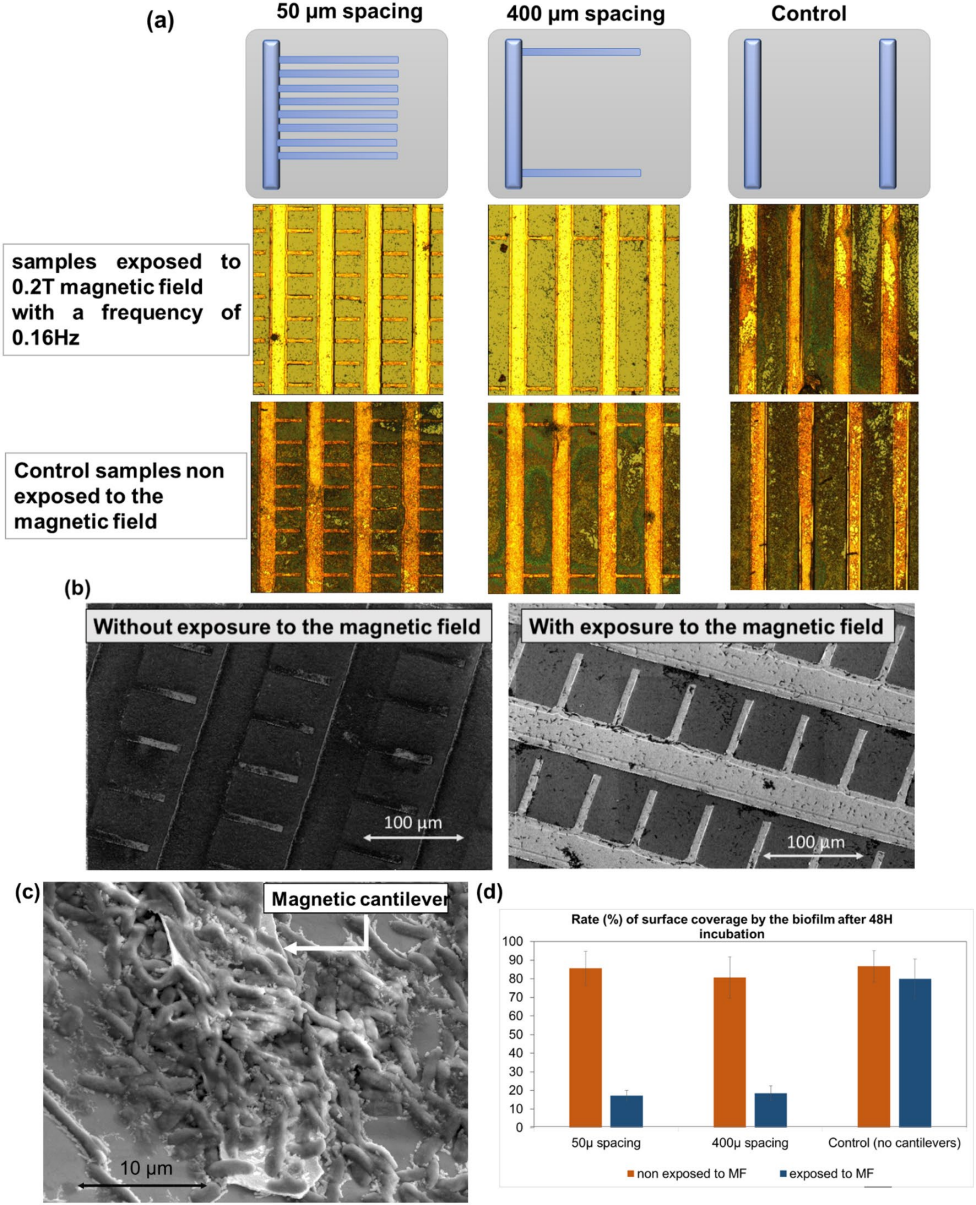
Biofilm formation on *E. coli* MG1655 inoculated substrates after 48 h incubation. Substrates were grafted with cantilevers spaced 50 µm or 400 µm apart, with a control area (bars) having no cantilevers. (**a**) Optical microscopy images of samples in the presence, or absence of an 0.2 T magnetic field, with a frequency of 0.16 Hz, for 48 h. (**b**) SEM images of substrates grafted with cantilevers spaced 50 µm in the presence or absence of the magnetic field. (**c**) SEM image of a single cantilever from the control chip (non-exposed to the magnetic field) colonized with bacteria. (**d**) Quantification of the substrate surface coverage by the biofilm following 48 h incubation.

Figure 5 (Fig. 5a) shows the proportion of the substrate that was colonised by the bacterial biofilm for two different cantilevers spacing (50 µm and 400 µm spacing between cantilevers), as well as the controls (areas where cantilevers were depleted), in the presence or absence of a continuous oscillating magnetic field of 0.2 T at a very low frequency of 0.16 Hz.

On the control samples, the bacteria showed a substantial level of growth, resulting in the formation of a biofilm, as shown in Fig. 5b. Multiple microcolonies appeared to have coalesced into larger (macro)colonies, with a high density of bacterial cells tightly intertwined in extracellular secreted material as shown by SEM in Fig. 5c. On the contrary, only individual bacterial cells or small bacterial clusters/colonies were observed on the sample exposed to the magnetic field. The quantification of surface coverage by the biofilm demonstrated an important reduction of about 70% in the microbial colonization and biofilm formation as shown in Fig. 5d.

The biofilm surface coverage rate (assessed by ImageJ software and detailed in the methods section) reveals that actuated magnetic cantilevers largely reduce the bacterial adhesion on the abiotic surface compared to the controls (*i.e.* surfaces covered with arrays of magnetic cantilevers, inoculated with the same bacterial suspension, but non-exposed to the magnetic field). Similar rates were observed for the two geometries tested, with concordant results for the controls, where the cantilevers were depleted, both in the presence and absence of the magnetic field.

The analysis of the optical microscopy images and the biofilm surface coverage rate, shown respectively in Fig. 5a, d, reveals that the magnetic cantilevers oscillation efficiently prevents the bacterial colonization on the sample.

## Discussion

Biofilm formation is a complex mechanism initiated by bacterial adhesion to abiotic surfaces, and it is controlled by the interplay between multiple biological factors^22,23^. These interactions are highly dependent on the properties of the surface on which the biofilm is developing. Even though the critical step of bacterial adhesion is not yet fully understood, it is widely accepted that bacteria react to surface topography^24^ aside from the response to surface chemistry (*i.e.* surface charge and surface energy)^25^. Many strategies are being actively pursued to develop efficient bacteria-repellent surfaces.

To date, most biofouling studies have investigated the correlation between bacterial attachment and static surface topography^26^, and there is little in the literature describing engineered dynamic surfaces with embedded physical properties, such as the mechanically actuated surfaces described in this study. Levering et al*.*^12^ previously described the mechanical disruption of mature *Proteus mirabilis* biofilms grown on elastomeric substrate by applying an inflation-generated strain. The use of magnetism to tackle biofilm formation has been recently exploited with the use of iron oxides nanoparticles. Hwang et al.^14^ observed an effective biofilm elimination when iron oxide nanoparticles are pulled through the biofilm structure using a permanent magnet. Quan et al*.*^13^ described the use of magnetic nanoparticles that are magnetically forced to create artificial channels in *Staphylococcus aureus* biofilms to enhance antimicrobials penetration in the biofilm structure, and induce the bacterial killing. More recently, Gu et al.^15^ reported a reduction in biofilm formation on *Escherichia coli* (UPEC), *Pseudomonas aeruginosa*, and *Staphylococcus aureus* strains by remotely controlling the beating of micron-size pillars loaded with superparamagnetic Fe_3_O_4_ nanoparticles using an external electromagnetic field.

In a previous study, Anselme et al.^27^ have reported that neither hydrophobicity nor chemistry alone are sufficient to predict bacterial attachment and that dynamic liquid flow plays a decisive role in the primary contact of bacteria with a surface. From our experimental results, we hypothesise that the bacteria-repelling effect of the magneto-mechanically actuated cantilevers is attributed to the hydrodynamic forces mediated through the oscillation of remotely actuated magnetic cantilevers. Indeed, substrate deformation due to magnetic field exposure causes a displacement of fluids, which could play an important role in microbial colonisation on surfaces both in transporting bacteria toward surfaces and in their adhesion process. We report this rationale as a hypothetic explanation to our experimental observations, which requires further investigations in a follow-on study focused on microfluid dynamics. Our hypothesis is based on the work of Bottier et al*.*^28^, who reported that the fluid flow induced by ciliary beating at 10 Hz create a horizontal velocity up to 400 µm/s close to the cilia wall. We acknowledge that the cilia beating pattern is asymmetric compared to the magnetic cantilevers oscillation in this study.

Studies carried out by Cellini et al.^29^ and Obermeier *et a*l.^30^ reported some effects of magnetic field on biofilm formation. Cellini et al. demonstrated that the exposure of *E. coli* to a 50 Hz electromagnetic field acts as a stressing factor leading to phenotypical and transcriptional changes^29^. In a set of experiments detailed by Obermeier et al.^30^, the exposure of culture of *Staphylococcus aureus* to a low-frequency electromagnetic field (5 mT, 20 Hz) exhibited a significant growth inhibition of the bacteria. However, the mechanisms governing the effect of magnetic field action are not fully elucidated and diverging assumptions provided in the current literature should be considered as speculative. In our experiments, we only observed a decrease in biofilm formation in the presence of cantilevers motion. The exposure to the magnetic field alone did not influence biofilm formation as shown on the control samples.

The important decrease in biofilm formation due to the magnetic microstructure oscillations was further investigated theoretically by calculating the magnetically induced cantilevers deflection. The magneto-mechanical model confirms that the cantilevers array presented here generate important mechanical deformation response, easily detectable optically. Indeed, an important optical response, consequence of the cantilevers arrays deflection to the magnetic field both in air and immersed in liquid, was demonstrated by reflection optical microscopy.

Magnetic forces F_Z_ exerted on a cantilever depend on the applied magnetic field and its gradient, *i.e.* on the magnetic source. Magnets of various compositions and dimensions could be considered to exert different ranges of magnetic forces. Depending on the pursued application, it is possible to either locally create larger gradients, thus producing larger forces (magnetic tips for example) or exert larger forces at longer distances (such as the magnet used for this study)^31^. The magnet used in this study is considered as a good compromise for generating sufficient forces on the cantilevers in experiments involving requirements linked to liquid bacterial cultures. Indeed, the presence of a stage and the use of bacterial culture dishes means that the cantilevers cannot be in direct contact with the magnet surface, but instead are located at a certain distance from the magnet. Our calculations demonstrate that the maximum cantilevers deflection of about 90 nm, close to a tenth of the size of an individual *E. coli* bacterium, is reached at a distance of 3 mm above the surface of the used magnet, a distance which is pertinent for our experimental setup.

One can notice the presence of a few individual isolated bacteria on the samples with oscillating cantilevers. It is possible that a biofilm could form and colonize this surface in the long term, as in theory, a single isolated bacterium could give rise to a biofilm through multiple cell division cycles. To address this facet, further experiments will be required to assess different bacterial species, both in mono and mixed cultures. Further investigation where bacteria are cultured for extended periods of time as well as in culture conditions that mimic different environmental or clinical/infection settings would provide further understanding of the use of such mechanically actuated microstructures for the prevention of bacterial biofilms formation.

A promising prospect for medical and industrial setups would be the combination of both chemical and mechanical strategies for biofilm elimination^4,32^. For example, further studies focusing on the use of magnetic cantilevers to impair the biofilm cohesion would provide more insights on a prototype that enhances the diffusion of existing antimicrobials within the EPS matrix.

When combined with an optical readout setup such as the one described by Truong et al*.*^17^, our device could also provide a standardised tool for the in situ characterisation of the mechanical properties of biofilms. This is particularly relevant, given the recent acknowledgement of the absence of standardised methods to characterise the mechanical properties of biofilms^33^.

### Conclusion

Diffusion of antimicrobial agents through biofilms is often limited by the multicellular structure encapsulated within the EPS matrices. In this study, we have demonstrated that remotely actuated microstructures grafted on abiotic surfaces can efficiently limit bacterial cell attachment and prevent the build-up of bacterial biofilms.

Our technology fulfils an unmet need for biofilm removal by efficiently preventing the critical adhesion stage at the substrate/bacterial suspension interface. This advance, in one of the most active research fields in microbiology may find broad applications both in clinical and industrial settings. The top down fabrication process offers a lot of flexibility and could be easily adapted for fabrication into more complex geometric designs if required.

The setup proposed in this paper opens up the possibility of standardised mechanical characterisation of microbial biofilms where the optical reading of the mechanical deflection amplitude would be directly related to mechanical properties of any biofilm grown on the device.

## Methods

### Magnetic cantilevers fabrication

The free-standing magnetic cantilevers were developed by a top-down technique on silicon (Si) substrates. A Polymethyl methacrylate (PMMA) photoresist was deposited by spin-coating on a silicon wafer to form a 200 nm thick sacrificial layer. Then, a layer of negative tone photoresist Ma-N 1,410, was deposited by spin-coating and patterned by direct-write optical lithography (MicroWriter, Durham Magneto Optics Ltd). A 100 nm layer of CoFeB was deposited by sputtering on a 2 nm Tantalum (Ta) layer and terminated by Pt and Ta. The remaining Ma-N 1,410 photoresist was then lifted-off in isopropanol solvent and the PMMA coated substrate was isotropically etched by oxygen plasma etching (Plasma barrel etcher, 3 min, 80 W, 8*10^–2^ mBar).

### *E. coli* attachment and biofilm culture

*E. coli* K12 MG1655 were maintained in Luria-Bertanni broth with 20% (volume/volume) glycerol at − 80 °C. Cultures were grown on tryptic soy agar at 37 °C for 24 h. Subsequently, bacteria were subcultured in Luria–Bertani broth for 1 h at 37 °C. Briefly, 1 h-old cultures of planktonic cells at optical density of 0.3 at 600 nm, which corresponds to a concentration of 2.4 × 10^8^ colony forming units (CFU)/ml were incubated statically for 48 h at 30 °C on horizontally placed cantilever covered substrates. A first set of samples were exposed to the magnetic field whereas the others were not and served as controls. The chips were then retrieved and evaluated for bacterial attachment. An incubation time of 48 h was chosen since it was observed that this time point allowed bacteria to attach to the surfaces in sufficient numbers for a meaningful qualitative and quantitative assessment.

### Image acquisition and processing

Visualisation of biofilm structures was conducted with both scanning electron microscopy (SEM) and optical reflexion microscopy. Surfaces were retrieved at 48 h after immersion in the bacterial culture, rinsed three times to remove planktonic bacteria and loosely attached cells, followed by a fixation in 2.5% glutaraldehyde solution. All the solutions used for rinsing and fixing the bacteria on the chips were prepared with sterile phosphate buffered saline (PBS) solution in order to minimize changes in osmotic forces and to avoid the detachment of bacterial biofilm from the substratum. After a final rinse in MilliQ water, the chips were left to air-dry. Microscopic observations were performed with reflected light microscopy.

Images taken by optical microscopy were processed using the software Image J. Images were binarized using the “adjust threshold” function. Then the surface coverage defined as the fraction of black pixels (bacteria and biofilm) over the total pixels (black and white pixels) was calculated using the “area fraction” function. Nine images taken at various arbitrary locations per sample were used to perform the image analysis.

### Calculation of the magnetic force applied to the cantilever

In order to calculate the magnetic force F_Z_ exerted by the magnet on the cantilever) and to determine the cantilever deflection δ_max_ at the cantilever free extremity as a function of Z (distance magnet to cantilever along the OZ axis. We first need to evaluate the magnetic field in the vicinity of the magnetic source, particularly the field component B_Z_ along the OZ axis.

### Estimation of the magnetic field acting on the cantilevers

**Figure 6:**
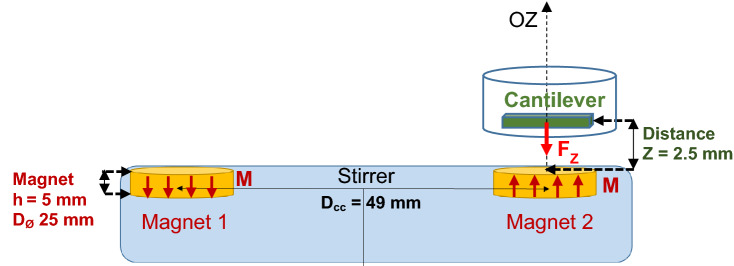
Sketch of the stirrer, showing a cantilever located above one of the magnets. Scheme including a cantilever (µm) and the two magnets (cm) not shown to scale: micrometric cantilevers actually quasi-punctual in the magnetic field generated by the magnet, along OZ axis. Dimensions, composition, field given as follows: (i) distance centre-to-centre between the two magnets Dcc = 49 mm; (ii) magnet characteristics: magnet thickness h = 5 mm (// OZ), diameter DØ = 25 mm; maximal magnetic field measured at the stirrer surface—i.e. at Z ≈ 1 µm -, B_Z_ =  + /—0.220 T: uniform magnetization M_MAG_ // OZ deduced from the magnetic field model: magnet µ_0_M_MAG_ = 1,18 T; (iii) measured distance from inside a well bottom to the stirrer surface, i.e. experimental distance Z from the magnet face to the cantilever, Z = 2.5 mm; (iv) cantilevers of composition Ta 2 nm Pt 10 nm CoFeB 50 nm Ta 2 nm CoFeB 50 nm Au 30 nm, thus total thickness h_CANT_ = 144 nm, and magnetic material (CoFeB) thickness h_MAGCANT_ = 100 nm; (v) cantilever dimensions 70 µm × 10 µm × 100 nm for the magnetic force F_Z_ calculation, total cantilever dimension 70 µm × 10 µm × 144 nm for the deflection calculation. Cantilever average Young modulus: a 1st hypothesis E ~ 200 GPa.

We consider a unique cantilever composed of Ta 2 nm Pt 10 nm CoFeB 50 nm Ta 2 nm CoFeB 50 nm Au 30 nm, placed above a stirrer magnet at a distance Z, on OZ axis of the cylindrical magnet face. Experimental set-up is sketched in Fig. 6.

The cantilever is an elongated parallelepiped, located at distance Z from the magnet face (dimensions in Fig. 2). The applied magnetic field and its gradient are considered as uniform over the whole volume of the cantilever due to the very small dimensions of the cantilever as compared to the magnet. The magnetic force exerted on the cantilever can be expressed as

3 $${F}_{Z}\left(Z\right)={V}_{magn}\cdot grad\left({\varvec{M}}\cdot {\varvec{B}}\right)$$

where **B** = µ_0_**H** is the field generated by the magnet, on the cantilever of magnetization **M**, located at distance Z from the magnet face, V_magn_ being the cantilever magnetic volume. Taking into consideration the magnetic properties of the cantilever (Ta _2 nm_ Pt _10 nm_ CoFeB _50 nm_ Ta _2 nm_ CoFeB _50 nm_ Au _30 nm_)—in particular the variations of the averaged magnetization M and of the differential susceptibility $\frac{dM}{d{B}_{Z}}\left(Z\right)$ as a function of the OOP applied magnetic field B_Z_(Z), generated by the magnet on OZ axis^34^, F_Z_ is expressed as follows^29^:

4 $${F}_{Z}\left(Z\right)={V}_{magn}\cdot \left(\frac{dM}{d{B}_{Z}}\left(Z\right)\cdot {\frac{d{B}_{Z}}{dz}\left(Z\right)\cdot B}_{Z}\left(Z\right)+M\left(Z\right)\cdot \frac{d{B}_{Z}}{dz}\left(Z\right)\right)$$

We consider here that the cantilever is magnetically saturated, *i.e.* B_sat_ ≤ B_Z_(Z) that is ${\mu }_{0}{M}_{\mathrm{CoFeB}}={\mu }_{0}{M}_{\mathrm{SATCoFeB}}= constant,$ and $d\left({\mu }_{0}M\right)/d\left({\mu }_{0}H\right)=0$ , with ${\mu }_{0}{M}_{\mathrm{SATCoFeB}}=1.25 T$ (M_SATCoFeB/Pt_ ~ 1,000 kA/m for instance in Ref^35^), the Eq. (1) is reduced as follows:

5 $${F}_{Z}\left(Z\right)={V}_{magn}\cdot \left({M}_{\mathrm{SATCoFeB}}\cdot \frac{d{B}_{Z}}{dz}\left(Z\right)\right)$$

For the determination of the magnetic field B_Z_ generated by the magnet on the cantilever and its derivative dB_Z_/d_Z_, we consider the magnet as a flat cylinder as described in Fig. 6, of thickness h = 5 mm and diameter D_Ø_ = 25 mm, generating B_Z_ along OZ. The magnet is modelled by the two surfaces of opposite charges, perpendicular to M_MAG_, separated by the distant h, each of them bearing a constant charge density σ =  + /−M_MAG_. Equations of B_Z_ (0,0,Z) and dB_Z_ /d_Z_ (0,0,Z) are described in Ref^31^. The circular section of the magnet is defined by 2A = 2B and (2A × 2B = π .(D_Ø_ ^2)/4). For the magnet used in our experiments: A = B = 11,078 µm; h = 5,000 µm.

By integration of the elementary fields generated by each unit of area, B_Z_ is analytically expressed at a point (X, 0, Z), as follows:

6 $${B}_{Z}\left(X,0,Z\right)=\left[{b}_{Z}\left(X,0,Z\right)-{b}_{Z}\left(X,0,\left(Z+h\right)\right)\right]$$

where

7 $${b}_{Z}\left(X,0,Z\right)=\left(\frac{{\mu }_{0}{M}_{MAG}}{4}\right)\cdot 2\cdot \left[{\mathrm{tanh}}^{-1}\left(\frac{\left(X+a\right)\cdot b}{Z\cdot \left(\sqrt{{\left(X+a\right)}^{2}+{b}^{2}+{Z}^{2}}\right)}\right)-{\mathrm{tanh}}^{-1}\left(\frac{\left(X-a\right)\cdot b}{Z\cdot \left(\sqrt{{\left(X-a\right)}^{2}+{b}^{2}+{Z}^{2}}\right)}\right)\right]$$

The gradient $d{B}_{Z}\left(0, 0,Z\right)/dZ$, required in the force calculation, derived from the expression of Bz:

8 $${dB}_{Z}\left(\mathrm{0,0},Z\right)/dZ=\left(\frac{{\mu }_{0}{M}_{MAG}}{4}\right)\cdot 4\cdot \left[g(Z+h)-g(Z)\right]$$

where

9 $$g(Z)=\left(a\cdot b\cdot \left({a}^{2}+{b}^{2}+{2\cdot Z}^{2}\right)\right)/\left(\left({a}^{2}+{Z}^{2}\right)\cdot \left({b}^{2}+{Z}^{2}\right)\cdot \left(\sqrt{{a}^{2}+{b}^{2}+{Z}^{2}}\right)\right)$$

**Figure 7:**
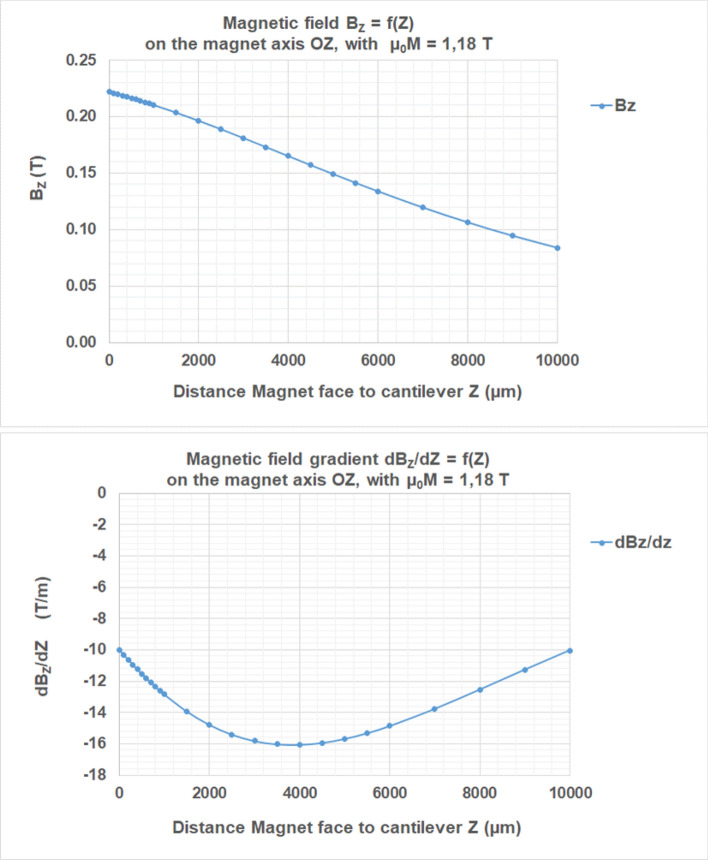
Resulting curves: magnetic field B_Z_ (0,0,Z) and dB_Z_/d_Z_, component of its gradient versus Z the distance magnet-face to cantilever. Z = 0 represents the magnet-face limit. Magnetization µ_0_M_MAG_ = 1.18 T. The applied magnetic field B_Z_ = 0,222 T; 0,22 T ; 0,189 T respectively at Z = 0 ; 200 µm and 2,500 µm.

The resulting B_Z_ and dB_Z_/d_Z_ f(Z) curves are shown in Fig. 7.

### Magnetic force applied on the cantilever.

**Figure 8:**
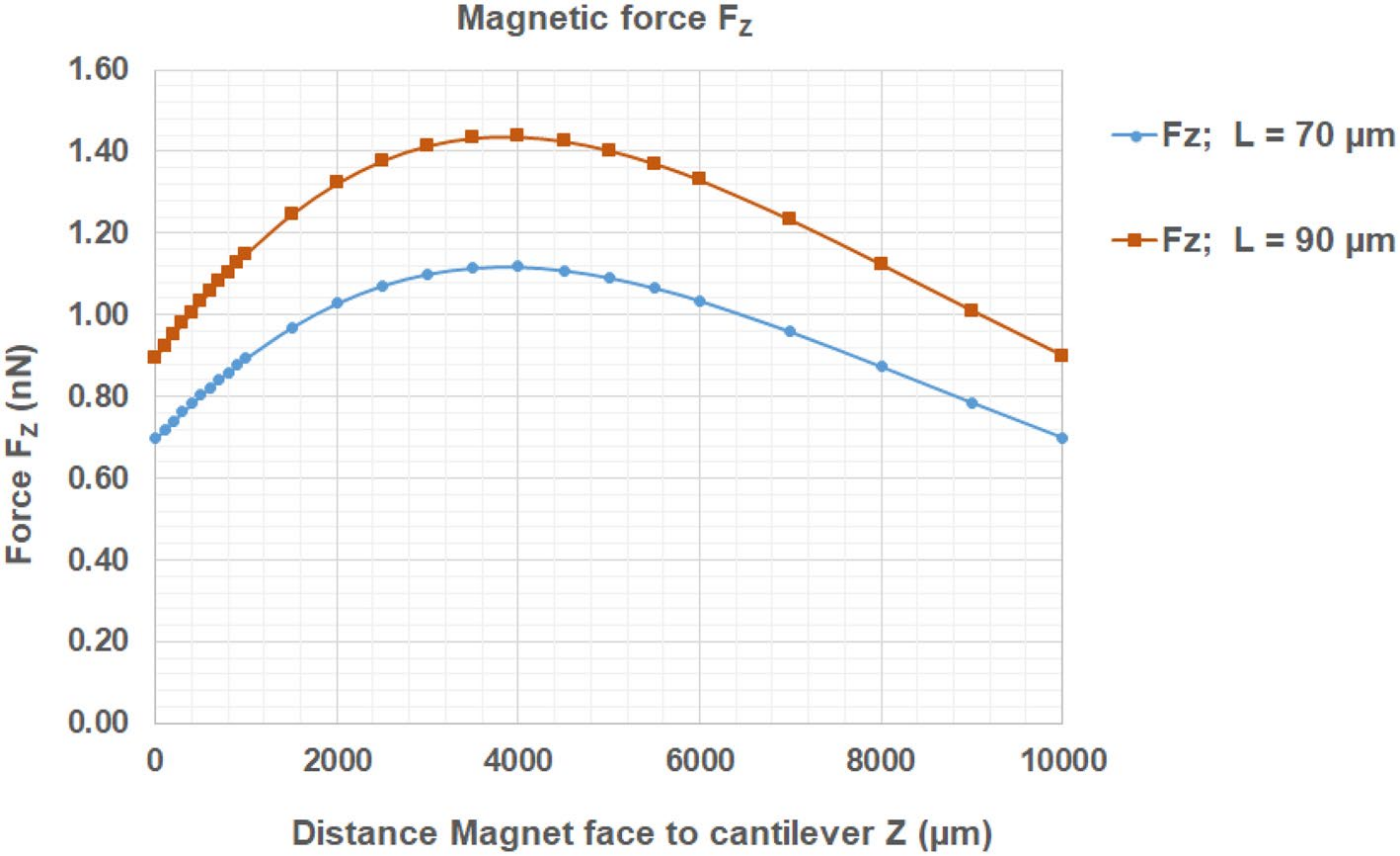
Magnetic force F_Z_ = V. M_SAT_dB_Z_/dz applied by the stirrer-magnet (magnetization 1,18 T) on the cantilever of magnetization µ_0_M_SAT_ (CoFeB) = 1,25 T, of length L, width LW = 10 µm, magnetic/(total) thicknesses: h_MAGCANT_ = 100 nm (h_CANT_ = 144 nm). Two lenths : L = 70 µm and L = 90 µm ; The force per unit length F_Z_/L is independent of the cantilever length L. At Z = 2,500 µm, F_Z_ = 1,07 nN and 1,38 nN respectively for L = 70 µm and L = 90 µm, and F_Z_/L = 1.53 × 10^–5^ N/m.

Considering the cantilever magnetization, ${\mu }_{0}{M}_{\mathrm{SATCoFeB}}=1.25 T$ , its magnetic volume (70 µm × 10 µm × 100 nm and 90 µm × 10 µm × 100 nm) , and the resulting $\frac{d{B}_{Z}}{dz}\left(Z\right)$ curve, the magnetic force applied on the cantilevers can be represented in Fig. 8.

The distance centre-to-centre between the two magnets is sufficiently large for neglecting the influence of the second magnet field on the cantilever located above the first magnet.

## Supplementary information

Supplementary Movie Legends

Supplementary Movie 1

Supplementary Movie 2
