# Supplementary material for: Magneto-mechanically actuated microstructures to efficiently prevent bacterial biofilm formation
Source: Sci Rep. 2020 Sep 22;10:15470. doi: 10.1038/s41598-020-72406-8 (PMC7508806; doi:10.1038/s41598-020-72406-8)
Supplement: Supplementary file 1 — Supplementary Movie Legends [file 41598_2020_72406_MOESM1_ESM.docx]

Supplementary information

**Magneto-mechanically actuated microstructures to efficiently prevent bacterial biofilm formation**

*Leulmi Pichot S* ^1^, Joisten H^2,4^, Grant AJ^3^, Dieny B^2^ & Cowburn RP^1^*

Corresponding author: [sl766@cam.ac.uk](mailto:sl766@cam.ac.uk)

1. Department of Physics, University of Cambridge, Cambridge, United Kingdom

2. Univ. Grenoble Alpes, CEA, CNRS, Spintec, 38000 Grenoble, France

3. Department of Veterinary Medicine, University of Cambridge, Cambridge, United Kingdom

4. Univ. Grenoble Alpes, CEA, LETI, 38000 Grenoble, France

# Legends for supplementary movies

Supplementary movie 1: Real time actuation under optical microscopy of magnetic cantilevers in air. The sample shows a reflectivity change when exposed to a magnetic field source (neodymium iron boron (NdFeB) magnet) as a result of the vertical deflection of the cantilevers array.

Supplementary movie 2: Real time actuation under optical microscopy of magnetic cantilevers in a Phosphate buffer saline (PBS) solution. The sample shows a reflectivity change when exposed to a magnetic field source (NdFeB magnet) as a result of the vertical deflection of the cantilevers array.
